# Screening for Heart Failure in Patients with Hypertension And/Or Diabetes Using Hand-Held Echocardiography: A Pilot Study

**DOI:** 10.5334/gh.1439

**Published:** 2025-06-19

**Authors:** Zi-Xuan Yang, Yu Kang, Xue-Ke Zhong, Qiao-Wei Chen, Yi She, Yun Guo, Xiao-Jing Chen, Hua Wang, Qing Zhang

**Affiliations:** 1 Department of Cardiology, West China Hospital, Sichuan University, Chengdu, Sichuan, China; 2 Tianfu New Area Zheng Xing Community Health Service Center, Chengdu, Sichuan, China; 3 Department of Emergency Medicine, West China Hospital, Sichuan University, Chengdu, Sichuan, China; 4 General Practice Medical Center, West China Hospital, Sichuan University, Chengdu, Sichuan, China; 5 Department of Cardiology, Beijing Hospital, National Center of Gerontology, Institute of Geriatric Medicine, Chinese Academy of Medical Sciences, Beijing, China

**Keywords:** Hand-held echocardiography, Heart failure, Community health service center, Screening

## Abstract

**Objective:**

This study aimed to assess the feasibility and cost-effectiveness of hand-held echocardiography-based screening for Stage B or C heart failure among individuals with hypertension and/or diabetes at the High-tech Area Fangcao Community Health Service Center and the Tianfu New Area Huayang Community Health Service Center in Chengdu, China, with the objective of promoting early diagnosis and intensified care.

**Methods:**

Patients with hypertension and/or diabetes registered and cared for at two community health service centers (CHSCs; Chengdu, China) with no history of clinical heart failure were recruited between October 2021 and December 2021. By combining symptom assessment (dyspnea and/or edema) and N-terminal-probrain natriuretic peptide (NT-proBNP ≥ 125 pg/ml as the cut-off) levels with HHE for any indexed abnormality in the dedicated semi-quantitative protocol, patients were categorized into heart failure (HF) Stages A, B, and C. The diagnostic accuracy and cost-effectiveness of several pre-specified screening strategies were compared.

**Results:**

Of the 423 patients (70 ± 9 years; males, 46.6%) enrolled, 166 (39.2%) were symptomatic and 106 (25.1%) exhibited elevated NT-proBNP levels. Hand-held echocardiography (HHE) abnormalities were detected in 286 (67.0%) patients, with interventricular septum thickening (47.0%) being the most common finding, followed by left atrial enlargement (30.0%). Left ventricular systolic dysfunction was identified in 18 (4.3%) patients. A total of 240 (56.7%) patients were reclassified as HF Stage B and 59 (13.9%) as Stage C. The stepwise strategy of using symptoms for initial stratification, followed by the selection of HHE or NT-proBNP in different circumstances, resulted in 100% accuracy and a 31.3% reduction in costs.

**Conclusions:**

HHE-based focused HF screening allows for the early identification of numerous cases of Stage B and mild Stage C HF in high-risk populations. A stepwise screening strategy incorporating symptoms, NT-proBNP, and HHE is feasible and cost-effective and should be adopted in community-based primary care settings.

## Introduction

With the growing epidemic of cardiovascular diseases, the burden of heart failure (HF) in China is increasing, approaching that of developed countries (1). It is important to control risk factors (stage A HF [SAHF]), treat asymptomatic cardiac structural and functional abnormalities (stage B HF [SBHF]), and delay progression to clinical HF (stage C HF[SCHF]) (2). Natriuretic peptides (NPs), the most commonly used biomarker for diagnosing HF and predicting outcomes, are effective in screening for asymptomatic left ventricular systolic dysfunction (LVSD) but exhibit a low sensitivity for other subclinical abnormalities, such as left ventricular hypertrophy (LVH) (3). Given its pivotal role in assessing cardiac structure and function, echocardiography is mandatory for HF screening. Compared with standard transthoracic echocardiography (sTTE), hand-held echocardiography (HHE) showed a comparable accuracy in measuring the left ventricular (LV) size, wall thickness, wall motion, and systolic function with easier access (4). Although it has been used in various clinical settings, it has not yet been incorporated into HF screening programs.

There are approximately 245 million individuals with hypertension (HTN) and 130 million individuals with diabetes mellitus (DM) in China (5). Under the national hierarchical medical system, several patients with uncomplicated HTN or DM received care from community health service centers (CHSCs) as part of chronic disease management projects. However, the management is inadequate, as reflected in numerous patients who neither know about their conditions nor receive appropriate treatment (6). Therefore, this pilot study aimed to investigate the prevalence of SBHF and SCHF among patients with HTN or DM (as SAHF) registered in the CHSCs and to demonstrate the feasibility of HHE-based HF screening strategies.

## Methods

### Study design

A cross-sectional study was conducted between October 2021 and December 2021 at the High-tech Area Fangcao Community Health Service Center and the Tianfu New Area Huayang Community Health Service Center in Chengdu, China.

### Study population

The participants were recruited from two CHSCs, who were patients aged ≥ 35 years with HTN and/or DM registered for chronic disease management. Patients were excluded if they had (a) a known history of HF, (b) a previous myocardial infarction, (c) a diagnosed structural heart disease, or (d) an unwillingness to participate. This study was approved by the ethics committee of West China Hospital. The study protocol was registered with the Chinese Clinical Trial Registry (ChiCTR2000041122). All the participants provided informed consent.

### Clinical assessment

Demographics, major comorbidities (including atrial fibrillation [AF], coronary artery disease [CAD], chronic obstructive pulmonary disease [COPD], and chronic kidney disease [CKD]), and chronic medications were collected from the records of the CHSC system. Body mass index (BMI), blood pressure (BP), and heart rate (HR) were recorded on the same day as the HHE. Meanwhile, the presence of dyspnea or edema was evaluated by fellows of cardiology under training according to a standardized questionnaire.

### Hand-held echocardiography

Transthoracic two-dimensional echocardiography with color Doppler was performed using commercial HHE equipment with a transducer (PHILIPS Lumify S4–1) and a monitor (HUAWEI MobilePad M5). By using a dedicated protocol, real-time scanning and semi-quantitative measurement were performed by an echocardiography fellow who was supervised by an attending cardiologist, in accordance with the guidelines of the American Society of Echocardiography (7). Two-dimensional and color Doppler images were acquired in the parasternal long-axis, parasternal short-axis, and apical views. The left ventricular end-diastolic dimension (LVEDD), left ventricular end-systolic dimension, interventricular septum thickness (IVS), and left atrial anteroposterior dimension (LA-ap) were measured. Pericardial effusion (PE), regional wall motion abnormality (RWMA), and valvular lesions were assessed semi-quantitatively.Impaired LV contraction was evaluated by both eyeballing and a left ventricular ejection fraction (LVEF) of <50%, calculated offline using the Teichholtz formula. In the case of uncertain or borderline results, the images were saved for further review by the supervisor.

HHE positivity was defined as any of the following abnormalities: (a) LV enlargement as LVEDD ≥ 55 mm (men) or ≥ 50 mm (women); (b) LA enlargement (LAE) as LA-ap ≥ 35 mm; (c) IVS thickening with IVS ≥ 12 mm; (d) RWMA; (e) moderate or severe valvular regurgitation; (f) valvular stenosis; (g) moderate or severe PE; and (h) LVSD as confirmed by offline-calculated LVEF < 50%.

### NT-proBNP assay

N-terminal-probrain natriuretic peptide (NT-proBNP) levels were measured using an electrochemiluminescent immunoassay (Roche Diagnostics) with a low detection limit of ≤5 ng/ml. The positive level was defined using a single cut-off of >125 ng/ml, irrespective of age, sex, or BMI.

### Stages of HF and interventions

Symptom assessment, HHE, and NT-proBNP testing were conducted simultaneously for every patient, and the derived HF stages, in accordance with the current guidelines were used as the ‘gold standard’ (Table 1) for testing the performance of the proposed screening strategies (2). Patients with LVSD in SCHF were defined as having HF with a reduced ejection fraction (HFrEF).

**Table 1:** Definition of HF stages.


SYMPTOM	HHE	NT-proBNP	HF STAGES	SUBGROUPS

–	–	–	SAHF	A1

+	–	–	SAHF	A2

–	–	+	SBHF	B1

–	+	–	SBHF	B2

–	+	+	SBHF	B3

+	+	–	SBHF	B4

+	–	+	SCHF	C1

+	+	+	SCHF	C2


HF, heart failure; HHE, hand-held echocardiography; NT-proBNP, N-terminal pro-brain natriuretic peptide.

For all participants, the status of HTN and/or DM control, as well as lifestyle modification, was reevaluated to adjust treatment if necessary. Patients with SCHF were referred to tertiary hospitals for a more comprehensive assessment regarding the confirmation of HF and its underlying diseases, complications, and comorbidities, followed by more intensified therapy. The majority of patients with SAHF or SBHF remained in management and follow-up at the CHSCs, where general practitioners (GPs) decided whether the patients needed more frequent check-ups based on abnormal findings. However, patients with symptomatic SAHF were recommended for the assessment of non-HF comorbidities. Asymptomatic patients with SBHF but HHE (+) and NT-proBNP (+) would also be referred for further evaluation if GPs deemed it necessary.

### Screening strategies

**Figure 1:**
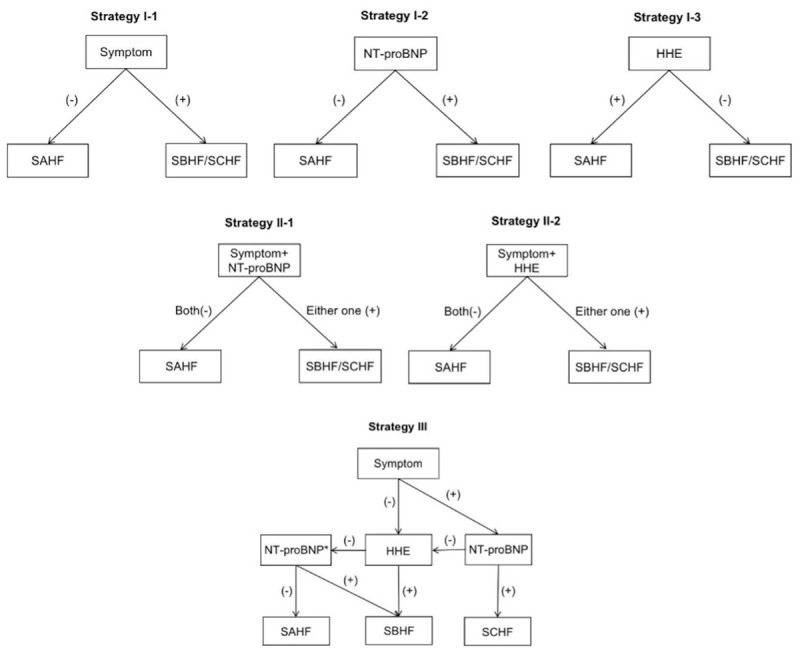
The scheme of HF stage screening strategies. HHE, hand-held echocardiography; NT-proBNP, N-terminal-probrain natriuretic peptide; SAHF, stage A heart failure; SBHF, stage B heart failure; SCHF, stage C heart failure. *If not performed/tested.

The prespecified screening strategies for SBHF and SCHF, including single tests (strategy I), combined tests (strategy II), and stepwise tests (strategy III) with different components, were proposed (Figure 1). Patients with at least one positive result were regarded as having SBHF or SCHF, as defined in Table 1. When compared with the ‘gold standard,’ the diagnostic accuracy and cost-effectiveness of each strategy were calculated.

### Statistics

Continuous variables were presented as means ± standard deviations or medians (interquartile ranges) when appropriate, whereas categorical variables were presented as percentages. One-way ANOVA, Mann-Whitney U, or chi-square tests were used for comparisons among groups with different HF stages. Statistical significance was defined as a two-sided p < 0.05. The diagnostic accuracy (sensitivity, specificity, negative [NPV] and positive predictive values, and accuracy) of the three screening strategies in reference to the ‘gold standard’ (Table 1) was expressed as a percentage with confidence intervals (CIs). CIs for sensitivity, specificity, and accuracy were ‘exact’ Clopper–Pearson confidence intervals. The CIs for the predictive values were the standard logit confidence intervals given by Mercaldo et al. (8).

### Cost-effectiveness analysis

The screening costs were estimated at ¥10.0 per participant for history and symptom assessment, ¥100.0 for the NT-proBNP test, and ¥100.0 for HHE (assumed to be 40% of a sTTE examination in a tertiary hospital). The cost per detected HF case for each strategy was determined by dividing the total cost by the number of identified SBHF and SCHF cases.

## Results

This study enrolled 423 patients (70 ± 9 years; 46.6% males). Overall, 155 (36.6%) patients had both HTN and DM, 203 (48.0%) had only HTN, and 65 (15.4%) had only DM. The durations of HTN and DM were 8 (2–15) and 0.3 (0–8) years, respectively. AF, CAD, COPD, and CKD were observed in 16 (3.8%), 55 (13.0%), 12 (2.8%), and 3 (0.7%) patients, respectively. The mean BMI was 25.2 ± 3.2 kg/m^2^. Overweight and obesity were observed in 190 (44.9%) and 73 (17.3%) patients, respectively. The mean systolic BP, diastolic BP, and HR on the day of screening were 135 ± 15 mmHg, 77 ± 10 mmHg, and 74 ± 14 bpm, respectively.

### Abnormal findings by clinical, NT-proBNP, and HHE assessment

A total of 166 (39.2%) patients had symptoms, including 41 (9.7%) with both dyspnea and edema, 68 (16.1%) with isolated dyspnea, and 57 (13.5%) with isolated edema. The average NT-proBNP level was 62.6 (34.2–125.2) pg/ml, and 106 (25.1%) patients were NT-proBNP (+).

HHE was feasible in all the patients. HHE (+) status was identified in 286 patients (67.0%). The most common positive finding was IVS thickening (47%), followed by LAE (30%). The coexistence of a thick IVS and large LA was observed in 65 patients (15.4%). LVSD by eyeballing was suspected in 9 (2.1%) patients but was confirmed by offline LVEF < 50% in 18 (4.3%) patients (69 ± 9 years; 66.7% males). The concordance of eyeballing with LVEF calculation was only observed in 6 of 21 patients (28.6%) who had been labeled as having LVSD by either method. Among the 15 patients with discordant LVSD, 12 patients without LVSD by eyeballing had an LVEF ranging from 43% to 49%, whereas three patients who were mistaken for LVSD by eyeballing had an LVEF between 50% and 59%. Moderate or severe valvular regurgitation was uncommon (8.7%), with tricuspid regurgitation having the highest incidence (5.2%). LV enlargement and RWMA were reported in 2.4% and 1.9% of patients, respectively. Notably, PE and valvular stenosis were not detected.

### Distribution of HF stages

**Figure 2:**
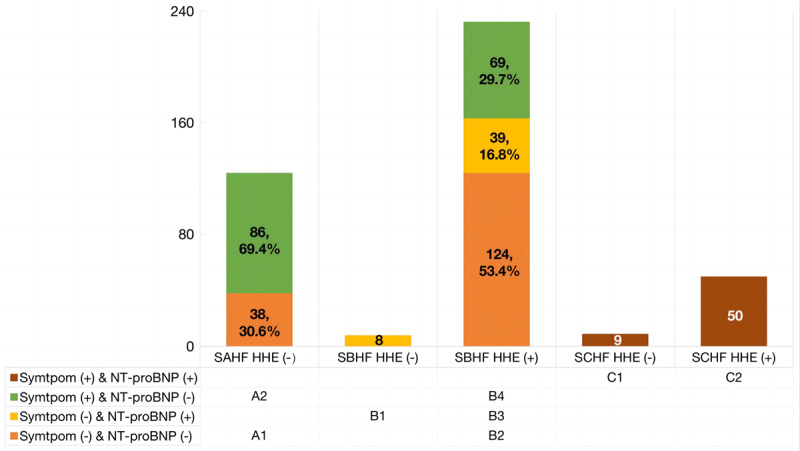
The distribution of HF stages and their subtypes. HHE, hand-held echocardiography; NT-proBNP, N-terminal-probrain natriuretic peptide; SAHF, stage A heart failure; SBHF, stage B heart failure; SCHF, stage C heart failure.

Post-screening, only 124 (29.3%) patients remained in SAHF, whereas 240 (56.7%) and 59 (13.9%) patients were found to have developed SBHF and SCHF, respectively. The subtypes of HF stages are shown in Figure 2. Among 18 patients with confirmed LVSD and a calculated LVEF of <50%, four patients with both symptoms and NT-proBNP (+) were labeled as having clinical HFrEF (only 6.8% of the patients with SCHF). In contrast, 11 patients who reported no symptoms (irrespective of NT-proBNP level) and three patients who had symptoms but a normal NT-proBNP level were regarded as having SBHF.

From stages A to C, the patients presented with a gradual increase in age, number of risk factors, and comorbidities, as well as NT-proBNP level, LA size, and IVS thickness, except that BP was the highest in Stage B (Table 2).

**Table 2:** Comparisons among HF stages.


	SAHF (n = 124)	SBHF (n = 240)	SCHF (n = 59)	P VALUE

Age, years	65.7 ± 9.9	70.2 ± 7.5*	76.1 ± 5.8*^#^	<0.001

Males, n (%)	46 (37.1)	122 (50.8)*	29 (49.2)	0.041

BMI, kg/m^2^	24.8 ± 3.2	25.3 ± 3.2	25.2 ± 3.6	0.353

SBP, mmHg	130.8 ± 17.1	137.4 ± 15.0*	134.4 ± 15.0	0.001

DBP, mmHg	76.4 ± 9.4	78.3 ± 10.0	74.8 ± 10.2^#^	0.030

HR, beats/min	75.5 ± 13.6	74.2 ± 14.3	73.2 ± 9.9	0.554

Hypertension, n (%)	93 (75.0)	210 (87.5)*	55 (93.2)*	0.001

Diabetes, n (%)	70 (56.5)	112 (46.7)	37 (62.7)^#^	0.040

AF, n (%)	1 (0.8)	7 (2.9)	8 (13.6)*^#^	0.001

CAD, n (%)	12 (9.2)	26 (10.8)	17 (28.8)*^#^	0.001

CKD, n (%)	0 (0.0)	1 (0.4)	2 (3.4)	0.073

COPD, n (%)	5 (4.0)	5 (2.1)	2 (3.4)	0.556

NT-proBNP, pg/ml	46.4 (28.7, 83.7)	58.7 (33.2, 103.5)	267.0 (175.8, 484.9)*^#^	<0.001

LVEDD, mm	42.5 ± 3.8	43.0 ± 5.3	43.9 ± 4.8	0.188

LA-ap, mm	29.2 ± 3.5	32.7 ± 5.4*	33.6 ± 6.7*	<0.001

IVS, mm	9.8 ± 1.0	12.2 ± 2.2*	12.4 ± 2.5*	<0.001

LVEF, %	67.4 ± 8.8	68.6 ± 10.1	67.6 ± 10.6	0.489


AF, atrial fibrillation; BMI, body mass index; CAD, coronary artery disease; CKD, chronic kidney disease; COPD, chronic obstructive pulmonary disease; DBP, diastolic blood pressure; HHE, hand-held echocardiography; HR, heart rate; IVS, interventricular septum; LA, left atrium; LVEDD, left ventricular end-diastolic dimension; LVEF, left ventricular ejection fraction; NT-proBNP, N-terminal pro-brain natriuretic peptide; SAHF, stage A heart failure; SBHF, stage B heart failure; SBP, systolic blood pressure; SCHF, stage C heart failure. *p < 0.05, compared with SAHF group; ^#^p < 0.05, compared with SBHF group.

### Diagnostic accuracy and cost-effectiveness of screening strategies

There were 299 patients with SBHF or SCHF identified by the ‘gold standard’, as shown in Table 1. In strategy I-1, patients with symptoms included 128/299 patients with Stage B or C and 38/124 patients with Stage A. The asymptomatic patients included 86/124 patients with Stage A and 171/299 patients with stages B or C. In contrast to strategy I-1, strategy I-2 had a lower sensitivity but a higher specificity with more missed diagnoses (193/299 vs. 171/299) but no erroneous diagnoses (0/124 vs. 38/124). Strategy I-3, compared to strategy I-2, had no missed diagnoses and showed the highest diagnostic accuracy among the single-test strategies. When NT-proBNP or HHE was used in combination with symptoms, both strategies II-1 and II-2 improved sensitivity at the expense of decreased specificity compared to their counterparts in strategies I-2 and I-3. Strategy III not only resulted in 100% sensitivity and specificity but also distinguished each HF Stage (Table 3). Furthermore, when compared to the ‘gold standard’, strategy III could save 31.3% of the cost (¥204.1 vs. ¥297.1) per case diagnosed with Stage B or C (Table 3).

**Table 3:** Diagnostic accuracy and cost-effectiveness of screening strategies.


	SINGLE TEST			COMBINED TESTS		STEPWISE TESTS	GOLD STANDARD TESTS
		
	STRATEGY I–1	STRATEGY I–2	STRATEGY I–3	STRATEGY II–1	STRATEGY II–2	STRATEGY III	

TP, n	128	106	282	175	291	299	299

FP/error diagnosis, n	38	0	0	38	38	0	0

TN, n	86	124	124	86	86	124	124

FN/missed diagnosis, n	171	193	17	124	8	0	0

Sensitivity (95% CI), %	42.8 (37.2, 48.6)	35.5 (30.1, 41.2)	94.3 (91.1, 96.7)	58.5 (52.7, 64.2)	97.3 (94.8, 98.8)	100	/

Specificity (95% CI), %	69.4 (60.3, 77.1)	100	100	69.4 (60.4, 77.3)	69.4 (60.4, 77.3)	100	/

NPV (95% CI), %	33.5 (27.8, 39.6)	39.1 (33.7, 44.7)	87.9 (81.1, 92.8)	41.0 (34.2, 47.9)	91.5 (83.9, 96.3)	100	/

PPV (95% CI), %	77.1 (69.8, 83.1)	100	100	82.2 (76.4, 87.1)	88.5 (84.5, 91.7)	100	/

Accuracy (95% CI), %	50.6 (45.7, 55.5)	54.4 (49.5, 59.2)	96.0 (93.6, 97.6)	61.7 (56.9, 66.4)	89.1 (85.8, 91.9)	100	/

Symptom cost*, ¥	4,230.0	0	0	4,230.0	4,230.0	4,230.0	4,230.0

NT-proBNP cost*, ¥	0	42,300.0	0	42,300.0	0	20,400.0	42,300.0

HHE cost*, ¥	0	0	42,300.0	0	42,300.0	36,400.0	42,300.0

Total cost, ¥	4,230.0	42,300.0	42,300.0	46,530.0	46,530.0	61,030.0	88,830.0

Cost per TP case, ¥	33.0	399.1	150.0	265.9	159.9	204.1	297.1

Cost reduction^#^, %	88.9	34.3	50.5	10.5	46.2	31.3	/


FN, false negative; FP, false positive; NPV, negative predictive value; NT-proBNP, N-terminal pro-brain natriuretic peptide; PPV, positive predictive value; TN, true negative; TP, true positive; * manifested as the number of cases tested multiplied by the unit cost; ^#^cost reduction = [1– (cost per TP case of each strategy/cost per TP case of gold standard)] × 100%.

## Discussion

In this pilot study, we aimed to implement HHE using a dedicated semi-quantitative protocol for community-level HF screening in high-risk patients. When combined with symptom assessment and NT-proBNP testing, over 70% of the population had previously known SBHF or SCHF. Owing to the limited medical resources and capacity at CHSCs, various screening strategies using a single test, combined tests, or stepwise tests have been proposed to evaluate their diagnostic accuracy and cost. It was suggested that the strategy involving initial stratification by symptoms, followed by HHE or NT-proBNP, resulted in the highest diagnostic accuracy while reducing test volume and costs.

### Impact of patients with Stage B or C HF in the community

The present study revealed a high proportion of SBHF or SCHF in patients with HTN or DM who were managed by CHSC as the grassroots of primary care in China. This was higher than the number obtained from a community population survey in Olmsted by the Mayo Clinic, which found that the burdens of Stage B and Stage C or D HF in adults ≥45 years were 34% and 12%, respectively (9). A similar prevalence (30% SBHF and 13% SCHF) was reported in 6118 participants (aged 67–91 years) of the Atherosclerosis Risk in Communities study (10). The progression of HF stages demonstrates not only clinical significance but also prognostic importance, which emphasizes the early detection of preclinical HF (11,12). The three-year risk of the composite outcome, including HF hospitalization, ischemic heart disease, stroke, and all-cause death, was 12.8%, 22.8%, and 31.8% for HF Stages A, B, and C, respectively (13). HF Stage-based approaches to prevent clinically overt HF are effective (14). BNP-based screening and collaborative care could reduce LV dysfunction and HF among patients with cardiovascular risk factors (15). Emerging therapeutic options in DM and intensive BP control in hypertensive patients can halt the progression of HF and reduce cardiovascular mortality (16).

### Promising role of HHE in HF screening

Recent meta-analyses have confirmed the prognostic significance of echocardiographic indices (including LVH and LAE) for adverse clinical outcomes in community-based epidemiological studies (17). Although echocardiography-based screening for asymptomatic Stage B or mild Stage C patients followed by new and more intensive treatment would become a valid preventive strategy (18), sTTE has not been recommended for HF screening and prevention in the community according to the current guidelines.

Image analysis of HHE achieved reasonable accuracy compared to that of sTTE for LV size, thickness, wall motion, and systolic function. Two decades ago, Galasko et al. first compared portable and traditional echocardiography devices for screening LV and valvular abnormalities in the community (19). The portable device had a sensitivity of 96% and a specificity of 98% for diagnosing LVSD. All cases of moderate or severe valvular regurgitation and 29 of 31 cases of significant LVH were correctly identified. The very high NPV saved costs for unnecessary sTTE, which would promote its use in community-based screening programs (19). The current study is the first to implement a dedicated HHE protocol that incorporates the measurement of dimensions and thickness, as well as semi-quantitative assessment of RWMA and valves. This focused screening approach by HHE in high-risk populations identified a substantial prevalence of LVH and LAE, which corroborated previous findings using conventional sTTE. Yang H et al. defined Stage B by LVH (13%) or LAE (31%) in a community population with HTN, DM, or obesity (20). Similarly, Wang et al. reported LVH in 23% and LAE in 35% of patients with DM aged ≥65 years in a community-based population (21). In addition, integrating HHE screening by primary care staff with remote interpretation by experts would be feasible to deliver more qualified care to low-resourced areas (22), whereas the development of automated quantification technology would be a potential avenue to improve reproducibility and reduce dependence on experts (23). Therefore, HHE offers another and probably better screening option that allows for easier access and lower costs.

### Screening strategy incorporated with symptom, NPs, and HHE

Dyspnea and edema are typical symptoms of HF, with fairly good sensitivities and specificities. They are used to distinguish clinical from subclinical HF in companies with NPs and echocardiography but fail to screen for SBHF. NPs are recommended for HF screening by guidelines; however, previous research demonstrated that NPs had very limited value in detecting LVH among patients with HTN and DM (24,25). NPs were the most accurate strategy for screening asymptomatic systolic LV dysfunction but inadequate for identifying SBHF (26). The cut-off of 125 pg/mL for NT-proBNP had a high specificity (>85%) but a low sensitivity (<35%) for echocardiography-detected subclinical HF. More evidence is needed to determine whether a lower cut-off for HF screening may yield a higher sensitivity (3).

In the recommended strategy III of this study, symptom assessment was adopted as the first stratification, followed by NT-proBNP and/or HHE. Those with symptoms and NT-proBNP (+) were SCHF who would not undergo HHE and were referred to sTTE, while those who were asymptomatic and HHE (+) were classified as Stage B who would not undergo NT-proBNP tests. Compared with other strategies tested, this stepwise strategy resulted in the highest accuracy in identifying a high-risk community population that progressed to HF stages B or C, as well as the ability to differentiate between the two stages. It was cost-effective compared to the ‘gold standard’ method of using the three components simultaneously.

However, there is no consensus on whether these screening strategies will eventually be integrated into real-life practice as an additional primary care evaluation in China. It relies on several major factors, such as financial resources, capacity to perform HHE in community settings, ongoing mentorship to ensure quality, and an established pathway of referral.

### Strengths and limitations

This cross-sectional study did not evaluate the impact of changes in therapeutic regimens based on the HF Stage. However, all patients were informed of their screening outcomes, and treatment or referral recommendations were provided according to the specific HF Stage. The current HHE device lacks spectral Doppler capabilities for the quantitative assessment of diastolic dysfunction and valvular abnormalities, highlighting the need for standardized training and long-term mentorship in the use of HHE. Although the use of a single cut-off as low as <125 pg/mL appeared reasonable in an HF screening study to ‘rule in’ suspected patients (i.e., high sensitivity), the NT-proBNP readings could be influenced by age, BMI, renal function, AF, and lung disease, potentially leading to false positive or false negative results. The lack of data on renal function or glucose control might affect further subgroup analysis.

## Conclusion

HHE-based focused HF screening allows for the early identification of Stage B and mild Stage C HF in populations with HTN and/or DM with a high detection rate. A stepwise screening strategy incorporating symptom assessment, NT-proBNP testing, and HHE appears to be feasible and cost-effective in community primary care settings.
